# Aptamers in Non-Small Cell Lung Cancer Treatment

**DOI:** 10.3390/molecules25143138

**Published:** 2020-07-09

**Authors:** Irena Wieleba, Kamila Wojas-Krawczyk, Paweł Krawczyk

**Affiliations:** Department of Pneumonology, Oncology and Allergology, Medical University of Lublin, 20-090 Lublin, Poland; kamilawojas@wp.pl (K.W.-K.); krapa@poczta.onet.pl (P.K.)

**Keywords:** aptamers, NSCLC, cancer

## Abstract

Aptamers are short, single-stranded oligonucleotides which are capable of specifically binding to single molecules and cellular structures. Aptamers are also known as “chemical antibodies”. Compared to monoclonal antibodies, they are characterized by higher reaction specificity, lower molecular weight, lower production costs, and lower variability in the production stage. Aptamer research has been extended during the past twenty years, but only Macugen^®^ has been accepted by the Food and Drug Administration (FDA) to date, and few aptamers have been examined in clinical trials. In vitro studies with aptamers have shown that they may take part in the regulation of cancer progression, angiogenesis, and metastasis processes. In this article, we focus on the potential use of aptamers in non-small cell lung cancer treatment.

## 1. Introduction

Nearly 85% of patients with lung cancer are identified as non-small cell lung cancer (NSCLC) cases. NSCLCs are divided into adenocarcinoma, squamous cell carcinoma, and large cell carcinoma [1,2]. The main treatment methods for NSCLC patients are radiotherapy, chemotherapy, immunotherapy, and targeted molecular therapy. Surgical resection takes place in only 15% of all NSCLC cases and most patients start their treatment in stages IIIB or IV. During these stages, cancer cells are progressing into epithelial–mesenchymal transition (EMT). Moreover, the neovascularization process begins and metastases to the local lymph nodes and distant metastases take place. However, the tumor microenvironment (TMI) is heterogeneous.

There are several genetic alternations in NSCLC which are mainly responsible for cancer initiation and development. Main genetic abnormalities occur in the *TP53*, *KRAS*, *EGFR*, *ALK*, *ROS1*, *BRAF*, *NRAS*, *RET*, *MET*, *NTRK1-3*, *FGFR1-4*, *KEAP1*, *STK11*, *NF1*, *SETD2*, *RBM10*, *MGA*, *ARID1A*, *PIK3CA*, *SMARCA4*, *RB1*, *CDKN2A*, *U2AF1*, and *RIT1* genes [3]. The major alterations mentioned above are responsible for the upregulation of the RTK/RAS/MAPK (Receptor Tyrosine Kinase/RAS/MAP Kinase) signaling pathway [3]. Routine diagnostic procedures commonly identify mutations in *EGFR* and *BRAF* genes and rearrangements in *ALK*, *ROS1*, and *NTRK1-3* genes [4]. These are predictable factors for treatment strategies in patients with non-resectable NSCLC in stage IIIB or IV [5].

The tumor microenvironment plays a main role in cancer development and is a specific factor for activation of tumor progression, epithelial–mesenchymal transition, and metastasis [6]. Typically, TMI features include high levels of immunosuppression and pro-angiogenic properties. Tumor cells have a poor surface antigen presentation and high expression of programmed-death ligand 1 (PD-L1) molecules [7].

Intercellular communication plays a main role in the EMT process, where the specific intercellular communicators are tumor-derived exosomes (TEXs). TEXs often include genetic material (mRNA, non-coding RNA, mtDNA, ssDNA, and dsDNA), regulatory peptides, and lipids. TEX has a place in the regulation of TME changes and providing appropriate conditions for metastatic processes [8]. Extracellular integrins form the TEX regulation system. Angiopoetins and metalloproteinases, such as VEGF, Angpt2, MMP3, and MMP10, activated by TEX are involved in the neovascularization process [9]. TEX exhausts natural killer cell functions by transforming growth factor beta 1 (TGF-β1), stimulates the transformation of monocytes into myeloid-derived suppressor cells (MDSC), and activates the suppression of cytotoxic T lymphocytes and apoptotic process of helper T lymphocytes [1]. Tumors work on a similar basis as a very advanced technological machine, where a great number of different processes are involved in the initiation, development, and progression of cancer.

In the group of patients in disease stages I–IIIA, surgical treatment or stereotactic radiotherapy are generally applied. For patients at more advanced stages of NSCLC, chemo/radiotherapy is the first-line treatment usually applied. Second-line treatment strategies depend mainly on the patient’s molecular profile and total health condition. Frequent brain metastases are a major problem. Targeted molecular therapy and immunotherapy enable significant improvement of treatment effects in oncological patients. Nevertheless, heterogeneous tumor structure, extensive metastases in the advanced stage of the disease, specificity of the intra-organ systemic distribution of the drug, and lack of assessment of micro-environmental factors in routine diagnostics contribute to unsatisfactory treatment results. There are still significant problems associated with severe adverse effects, drug resistance, non-satisfactory progression-free survival, and overall survival. There is a clear necessity for developing therapies enabling the complete recovery of locally advanced and advanced cancer patients. In view of the above, aptamers may have great potential for targeted molecular therapies in cancer patients.

## 2. Pharmacokinetics and Biological Safety of Aptamer Use

**Figure 1:**
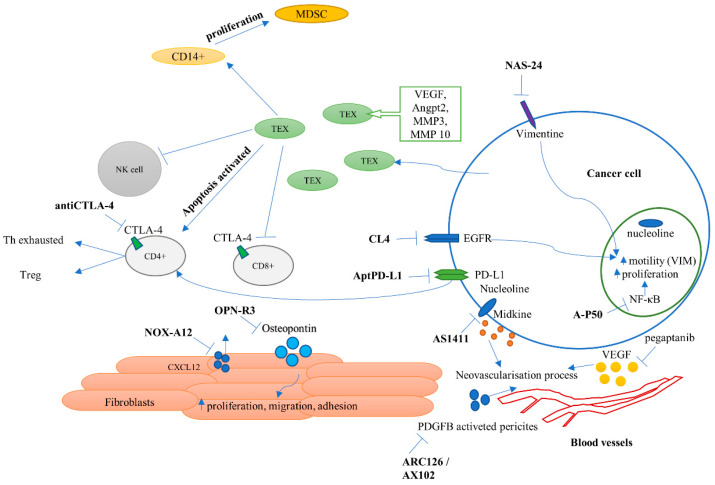
Molecular targets and biological role of mentioned aptamers in the cancer microenvironment.

The notion of “aptamer” is derived from the Latin word “aptus”, which means “adapted” or “conformable”. Nucleic aptamers are single-stranded RNA or DNA oligonucleotides which show high affinity and binding specificity for organic compounds (including proteins) or inorganic molecules. Aptamers have been known for about 30 years. However, only one aptamer has been registered as a drug (pegaptanib) to date, while another aptamer is close to registration (mipomersan). At present, 43 clinical trials can be found on the use of aptamers to treat various diseases, mainly macular degeneration, diabetes, leukemia, and solid tumors. Aptamers, as relatively small-sized oligonucleotides, present several challenges for successful clinical translation. Since the approval of the first aptamer drug, Macugen^®^, by the FDA for patients with age-related macular degeneration, several aptamers have been developed which showed promising anticancer effect in pre-clinical models, as well as in clinical trials. In recent years, the interest of researchers in these types of compounds and their potential in cancer therapy has clearly increased. Figure 1 presents the main intracellular and extracellular targets for aptamers which can be used in the treatment of lung cancer [10]. The pictured aptamers and their modifications, such as bifunctional chimeric particles and nanocarriers, are described in this article.

**Figure 2:**
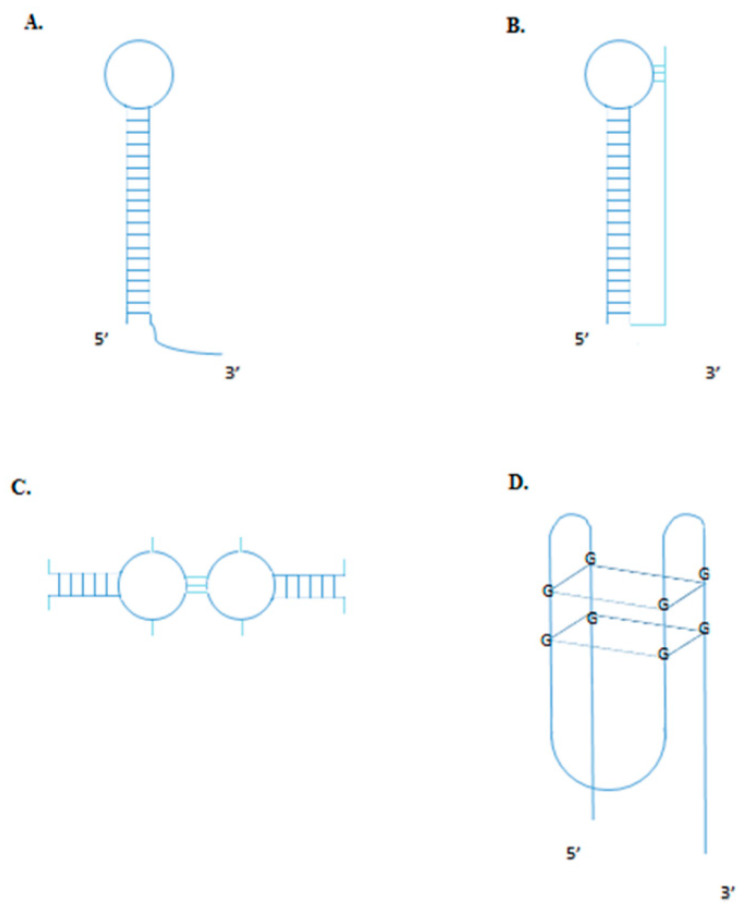
Aptamer secondary structures: (**A**) Hairpin motif; (**B**) Pseudoknot motif; (**C**) “Kissing loops” motif; and (**D**) G-quadruplex chair folding structure.

**Figure 3:**
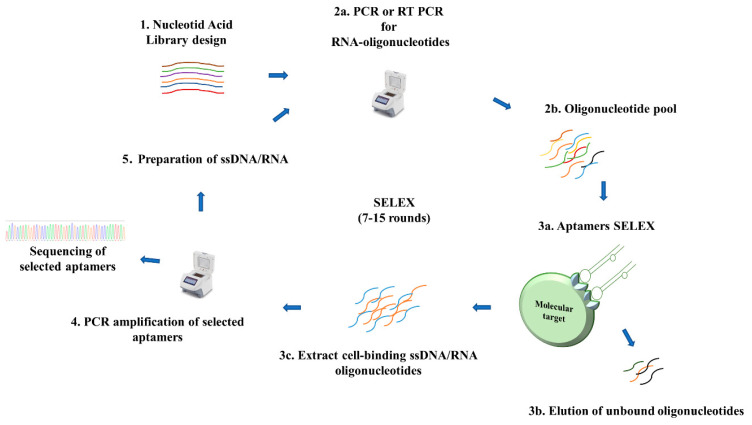
Aptamer design and selection (SELEX: Systematic evolution of ligands by exponential enrichment). The main steps of SELEX are: (**1**) Initial library design; (**2**) Preparation of the target molecule and selection (a. nucleotide synthesis by PCR; b. in this step, post synthesis chemical modifications are possible); (**3**) Separation of non-specific from specific-binding molecules (a. aptamer incubation with molecular target; b. elution of unbound oligonucleotides; c. extraction of specific-binding aptamers); (**4**) Amplification of selected sequences in PCR; and (**5**) Preparation of ssDNA/RNA.

Aptamer acts as ligands, binding very strongly to a target molecule. The dissociation constant K_d_ is used to describe the binding strength (or affinity) between receptors and their ligands. The dissociation constant (K_d_) determines whether the target molecule–ligand complex is stable. To be useful, aptamers should have K_D_ values in the pico- to nano-molar range. For example, the dissociation constant for cetuximab (anti-EGFR antibody) is 2.3 nM, while the K_d_ of the fluorine-modified (2′-fluoropyrimidine) anti-EGFR aptamer is comparable, equal to 2.4 nM. Aptamers are typically stable and have low variability during the production process [11]. Although their molecular weight is typically relatively small—approximately one-tenth that of monoclonal antibodies—their complex tertiary folded structures create sufficient recognition surface area for tight interactions with their target molecules. Figure 2 presents functional secondary structures of aptamers. The DNA hairpin motif acts with proteins involved in replication and genome translocation and the RNA hairpin motif acts as binding site for proteins, thus being mainly engaged in enzymatic reactions. The RNA hairpin structural motif is more compact and is maintained by base pairing in the stem region, whereas the hairpin loop in DNA has less conformational variations and is conditioned by the polymer’s formality. Hence, RNA aptamers with hairpin structural motifs may have better affinity and stronger interactions with enzymatic proteins [12]. Pseudoknots are RNA structural motifs involved in gene regulation and protein synthesis processes. Pseudoknots are variable structural motifs, which can easily take on the form of a 3′- or 5′-hairpin structural motif. Their stability is predicted by the presence of a ligand. Interactions between pseudoknots and different molecules involved in the regulation of gene expression have been confirm [13]. “Kissing loops” are formed between the stem loops of nucleic acids without free 5′- or 3′-ends. This structural motif might be useful in nanotechnology, especially for nanocarrier synthesis [14,15]. G-quadruplex is an RNA and DNA structural motif involved in epigenetic regulation and mtDNA regulation. Its potential in cancer development and treatment has been the subject of many studies in recent years. The G-quadruplex motif has different folding structures, as determined by the strand direction and glycosidic conformation: parallel, intramolecular hybrid, basket, and chair folding structures. The chair folding structure is presented. Guanine-tetrads form a square planar platform held by Hoogsten hydrogen bonds [16,17]. G-quadruplex structures have high stability and lack sensitivity to nucleases. Hence, aptamers with the G-quadruplex motif may have a longer plasma half-life. Additionally, unlike antibodies, aptamers can be readily chemically synthesized and modified [18]. In addition, the long storage period and low immunogenicity of aptamers are favorable properties for clinical utility. Due to their flexibility in terms of chemical modification, aptamers can be conjugated to other chemical entities including chemotherapeutic agents, siRNA/miRNA/plasmid shRNA, and nanoparticles for therapeutic and diagnostic applications. After the systematic evolution of ligands by exponential enrichment (SELEX) protocol was first published in 1991, many methods modifying the classical SELEX method have been published. There have been several reviews on the technical aspects of aptamer synthesis and applications. Two in particular seem to be good analytical reviews pointing at the knowledge gathered from that time: Odeh et al. described the chemical modification possibilities for therapeutic aptamers, highlighting the possible adverse effects [19], while Wang et al. analyzed the whole technological process of aptamer synthesis. They also described novel bioinformatical tools for designing and predicting aptamer structures and their interactions with a given target [20]. The principles of the SELEX method are presented in Figure 3.

The half-life of chemically unmodified aptamers in the body is only a few minutes [11]. Their short plasma half-lives due to nuclease degradation and rapid renal excretion have necessitated further structural modifications for useful clinical application [21]. Hydroxylation of the ribose residue resulted in an increase of aptamer serum half-life from several minutes to several hours or days. To make aptamers unsusceptible to nucleases, the 5′ and 3′ ends of the oligonucleotide chain have been modified or so called ‘mirror’ aptamers (spiegelmers) have been synthesized, which contain L-deoxyribose. For example, the half-life of the TS-1 aptamer, which has ‘hairpin’ structures and modifications at its 5′ and 3′ ends, is up to 50 h. To avoid the rapid excretion of aptamers by the kidneys, post-synthesis chemical modifications are used. Some popular examples are: pegylation (covalent binding of polyethylene glycol to aptamer), the addition of cholesterol and peptides, and multimerisation (e.g., homodimerization) [18]. For example, the first aptamer registered by the FDA (Food and Drug Administration), pegaptanib (Macugen^®^), presents these modifications. The side chain of pegaptanib is connected to polyethylene glycol (PEG). The half-life of pegaptanib in human serum is 9.3 h [11,18,21].

Pegaptanib is a vascular endothelial growth factor (VEGF) antagonist. It has been approved in the USA and the European Union for the treatment of patients with age-related exudative macular degeneration (AMD) caused by abnormal blood vessel growth. Pegaptanib used for intravitreal injections may cause anterior chamber inflammation, eye pain, increased intraocular pressure, punctate keratitis, and vitreous opacity. Pegaptanib as an anti-angiogenic drug has potential use in the treatment of cancer. Macugen^®^ administered intravenously did not show significant toxicity in monkeys. However, in a phase II clinical trial, eight out of 13 cancer patients showed adverse reactions related to the intravenous administration of this pegylated aptamer [11,18,21].

A majority of clinical trials involving aptamers are confirmed/withdrawn in stages I/II, respectively, probably due to the low stability of aptamers and the several side effects caused in patients. Table with clinical trials of selected aptamers mentioned in this article is added to the Supplementary Material. Pre-clinical and clinical trials have shown that aptamers accumulate in the liver, kidneys, and spleen. First- and second-phase clinical trials of mipomersan (Kynamro^®^, apolipoprotein B synthesis inhibitor) in patients with familial hypercholesterolemia showed a temporarily increased level of hepatic enzymes, depending on the aptamer dose (30–400 mg/kg/week), during five weeks of treatment. In phase III clinical trials, a three times higher level of alanine transaminase (ALT) in 17.9% of patients after 26 weeks treatment was observed [22]. In March 2013, the European Medicines Agency (EMA) rejected marketing authorization of mipomersan, due to the potential long-term side effects of the drug, as expressed by abnormal liver function tests showing fat accumulation and elevated liver enzymes in the blood. This review discusses the advantages and challenges of aptamers and introduces the therapeutic aptamers currently under investigation in clinical trials in cancer patients [11].

## 3. Aptamers in NSCLC Treatment

One of the most frequently studied aptamers is AS1411, which has a G-quadruplex structure. G-quadruplexes are short, four-stranded nucleotide structure formed in DNA or RNA, which have a high guanine content. The presence of such G-quadruplexes in human cells has been demonstrated under natural conditions. They are responsible for the additional stimulation of promoter regions of various genes (e.g., *c-MYC*, *VEGF*, *HIF-1α*, *BCL-2*, *K-RAS*, *PDGF-A*, and *PDGF-Rβ*) and are involved in telomerase shortening. Earlier studies have shown the potential anti-tumor activity of G-quadruplexes, their participation in cell cycle regulation, and regulation of cell proliferation [23]. G-quadruplexes are potentially interesting structures for therapeutic aptamer discovery, based on their determined regulatory functions on oncoprotein expression and their hypothetical lower toxicity.

AS1411 specifically binds to nucleolin, a phosphoprotein which is highly expressed on the cell surface of different cancer sub-types. The connection of AS1411 with nucleolin inhibits the intracellular reaction between nucleolin and the mRNA of *BCL-2*. Destabilization of *BCL-2* mRNA decreases synthesis of the Bcl-2 protein, which has strong anti-apoptotic properties. Nucleolin is also involved in processes associated with the cell cycle, angiogenesis, and the production of cytokines and pro-inflammatory factors [16,17]. It also acts as a receptor for TNF-α (tumor necrosis factor alpha) and stimulates the synthesis of TNF-α and NF-κB [24]. NF-κB is a transcription factor which is responsible for activating the transcription of several genes involved in cell proliferation, inflammation processes, and immune response [25]. The occurrence of nucleolin on the surface of cancer cells has been associated with the activities of VEGF and MMP9 (matrix metalloproteinase 9). This indicates the involvement of nucleolin in the epithelial–mesenchymal transformation process [26]. Extracellular nucleolin has also been shown to be associated with the internalization of specific ligands. One of the specific surface ligands on lung epithelial cells is midkine, which belongs to the growth factors family and is responsible for heparin binding to the cells [26,27]. It is also involved in the processes of angiogenesis and metastasis in lung cancer. Midkine occurs at high concentrations in pleural fluid in patients with advanced NSCLC, which is an unfavorable prognostic factor in this disease [27,28]. Therefore, the anti-nucleolin AS1411 aptamer may play an important role in the inhibition of tumor progression and metastases through enhancing the EMT process.

In cell cultures including A549 (human lung adenocarcinoma), the addition of AS1411 has been shown to produce cell cycle arrest in S phase by blocking DNA replication [26,29]. Reyes-Reyes et al. discovered that AS144 induced the phosphorylation of AKT and Rac1 protein in A549 cells. Rac1 activation may be partially mediated by EGFR activation. Anti-EGFR monoclonal antibody treatment inhibited the antiproliferative function of the aptamer only at very low doses of AS1411. AS1411 treatment induced prolongated activation of Rac1, which mediates micropinocytosis. Intensive micropinocytosis, a clathrin-mediated endocytosis, may involve methuosis [29]. Therefore, the anti-nucleolin AS1411 aptamer may play an important role in the inhibition of tumor progression and metastases through the EMT process, but investigation of the AS1411-mediated regulatory pathways is needed.

Hombloe at al. tested intracellular absorption of radioactive labelled AS1411 aptamers (57Co-DOTA-AS1411) by non-small lung cancer stem cells (CSC). For this purpose, CSC cell spheroids were obtained from A549 and H1299 cell lines. Intracellular uptake was 4–5 times higher for CSC cell cultures than for non-CSC cell cultures. Moreover, 57Co-DOTA-AS1411 was accumulated in the cell nucleus, as a high level of intracellular nucleolin formed [30].

Alibolandi et al. created nanopolymersome loaded by gemcitabine Apt-GEM-NP. The main core of this nanocarrier consisted of polyethylene glycol-poly(lactic-co-glycolic acid) decorated with the aptamer AS1411 for selective drug delivery into the nucleolin-positive NSCLC cell. In vitro tests on the A549 cell line showed receptor-mediated transport and effectiveness of the selective therapy compared to the control [31].

Ayatolllahi et al. developed a PAMAM (polyamidoamine)-based platform decorated with AS1411 for selective delivery of the sh-RNA plasmid into A549 cells. PAMAM was conjugated with 10-bromodecanoic acid and 10C-PEG for a more relevant transfection rate. Plasmid sh-RNA inhibited Bcl-XL expression through regulation of miRNA levels. The expression level of Bcl-XL was 25% [32].

Askarian et al. developed a polyplex complex consisting of poly(l-lysine) (PLL) and polyethylenimine (PEI) conjugated with the aptamer and plasmid shRNA for downregulation of Bcl-XL expression in lung cancer cells. The complex size was less than 128 nm. In vitro tests showed efficient transfection rates and low toxicity levels [33].

Pegaptanib (26-nucleotide anti-VEGF165 aptamer) inhibits the angiogenesis of blood vessels. It is specific to VEGF_165_, a glycosylated homodimeric VEGF isoform which causes pathological vascular formation within the tumor, increased permeability of blood vessels, and inflammation (via production of IL-1, IL-8, and TNF-α). VEGF_165_ is one of the factors responsible for the development of age-related macular degeneration [34]. In cancer patients (also in colorectal cancer), the main factor responsible for tumor neoangiogenesis is another isoform of vascular endothelial growth factor, VEGF_121_. In these patients, an increased concentration of the VEGF_165_ isoform in the course of tumor development may be associated with vascular maturation. In contrast, in patients with ovarian cancer, high concentrations of VEGF_165_ may be responsible for neovascularization of the tumor tissue [35]. Therefore, the use of pegaptanib may not be justified in all types of cancers. However, in preclinical studies in cells culture (A549) and in xenograft mouse models with lung adenocarcinoma, pegaptanib in combination with a cotinine-specific antibody showed inhibition of the angiogenesis process and reduction of tumor growth, in comparison to the (untreated) control group. The effectiveness of pegaptanib was comparable to the results obtained with bevacizumab (anti-VEGF antibody), which has been registered for NSCLC patient treatment (in combination with first-line chemotherapy) [2].

ARC126/AX102 is an aptamer specific to the B sub-unit of platelet-derived growth factor (PDGFB). PDGFB acts on pericytes and is responsible for their recruitment into emerging blood vessels within the tumor [36,37]. Preclinical in vitro and in vivo studies confirmed the anti-angiogenic effect of ARC126/AX102 in lung cancer [38].

The frequency of *EGFR* (epidermal growth factor receptor) gene mutations in NSCLC patients is around 10–14% in Caucasian patients and exceeds 40% in Asian patients [4,26]. In patients with stage IIIB or IV NSCLC and with activating mutations in the *EGFR* gene (in exon 18–21), EGFR tyrosine kinase inhibitors (TKIs) have been successfully used [39,40]. Unfortunately, during the treatment of EGFR TKIs, cancer cells can become resistant to such therapy. Therefore, research on new molecules which can break the resistance of cancer cells to EGFR TKIs is of great interest. Such molecules may include EGFR-specific aptamers. One of them is the RNA aptamer E0727/CL428/KD1130/TuTu223, which can inhibit the excessive proliferation of cancer cells and initiate the intracellular pathway for activating apoptosis in cell cultures with activated *EGFR* gene mutations. Aptamers E0727, A1532/4233, and CL434 also have the ability to inhibit EGFR autophosphorylation in cancer cells [38,41].

CL4 is a 39-nucleotide 2′-fluoropirimidine-modified EGFR-specific RNA aptamer. Studies have shown the short half-life of CL4 in blood serum, which limits its use in clinical practice. However, Wang et al. developed a new aptamer called CL-4RNV616 based on the CL4 sequence. CL-4RNV616 constitutes 27 chemically modified RNA and DNA nucleotides. In the ribonucleotides, a hydrogen atom in the hydroxyl group (2′-OH) was replaced with a methyl group (2′-O-CH_3_) at the second carbon atom of the sugar residue. The presence of the methyl group contributed to higher aptamer compatibility with EGFR, lower toxicity, and significantly higher stability of the complex. The dissociation constant of CL-4RNV616 is about 100 nM, which indicates its high affinity to EGFR. In vitro studies of CL-4RNV616 showed strong pro-apoptotic effects on selected tumor cell lines differing in EGFR expression. In materials derived from human breast tumor biopsies, Wang et al. observed a higher affinity of CL-4RNV616, compared to anti-EGFR antibodies, to the EGFR molecule [41].

Parassieli et al. developed a chimeric molecule of CL4 and ipilimumab, an anti-CTLA-4 monoclonal antibody. To obtain the CL4–ipilimumab complex, the monoclonal antibody had hydrazinonicotinamide incorporated into the FC region and was conjugated to the aptamer by a chemical linker. The CL4–ipilimumab complex was tested on EGFR+ cancer cell lines and an EGFR/CTLA4-positive SK-BR-3 cell line. Cell proliferation inhibition for monotherapy was nearly 20%, while the inhibition score for the chimera was nearly 40%. Studies have also examined the lymphocyte activation activity of the chimera complex, compared to the control (monotherapy by CL4 or ipilimumab). The highest levels of interleukin-2 and interferon-γ were detected with the CL4–ipilimumab complex [12].

The AP/ES (anti-EGFR/erlotinib and surviving-shRNA) delivery system, in complex with chloroquine, was developed by Lv et al. for selective delivery of erlotinib and surviving-shRNA into erlotinib-resistant non-small cell lung cancer tumors. Selective delivery was reached by the anti-EGFR aptamer conjugated with polyamidoamine. Chloroquine plays a role in escaping from endosome lysis and stimulating tumor microcirculation [42].

Li et al. developed a liposomal complex attached to an anti-EGFR aptamer conjugated with chitosan for specific delivery of erlotinib and perfluorooctyl bromide. In vitro and in vivo tests showed it significant role in the normalization of hypoxia and inhibition of tumor growth [43].

GL.21.T is a 2-fluoropyrimidine-modified RNA aptamer developed by Nuzzo et al. The authors also created the GL.21.T form conjugated with miRNA-137, which was called AmiC. The study was conducted in an autologous NSCLC cell culture and in xenograft mouse transfected with erlotinib-resistant A549 cells. Paclitaxel was used for control group treatment. MiRNA-137 downregulates the expression of Axl (a tyrosine kinase receptor). The overexpression of Axl has been correlated with higher activities of transcription factor NF-κB and MET kinase, as well as the occurrence of *T790* mutation [44]. Several studies have confirmed direct relationships between the overexpression of Axl and drug resistance to erlotinib, gefitinib, or osimertinib [45]. GL.21.T inhibited cancer cells migration in 75%, while the aptamer complex with miRNA inhibited in 80%. A single aptamer has no antiproliferative activity. AmiC inhibited tumor cell growth in 30% after 72 h incubation. The researchers obtained a comparable result in the inhibition of viability for paclitaxel and AmiC. The intracellular level of miRNA-137 after treatment with the AmiC complex was closer to the physiological level than in cells transfected only by miRNA-137 [11].

Russo et al. created the GL21.T-miR-34c chimera for selective delivery of miRNA-34c to Axl+ non-small cell lung cancer cell lines. Bioinformatic analysis and in vitro tests showed that the intracellular level of miRNA-34c was significantly lower in NSCLC cells than in normal lung tissue. Calu-1 cell transfection by the GL21.T-miR-34c complex inhibited cell proliferation and cell migration by 20%. The GL21.T-miR-34c complex also increased cell sensitivity to radiotherapy and overturned sensitivity to erlotinib in an erlotinib-resistant cell line. The intracellular transport of GL21.T-miR-34c complex is receptor-dependent. The serum stability of the GL21.T-miR-34c chimera is about 8 h [46]. The GL21.T-miR-212 chimera was also created, where MiR-212 stimulates tumor sensitivity to TRAIL treatment through a decrease of PED level [47].

Kim et al. created gold nanoparticles marked by aptamers for the specific delivery of a functional peptide to lung cancer cells [48].

Wang et al. created an aptamer-labelled nanocarrier PAM-Ap/pMiR-34a, which is a dendrimer conjugated with PEG and an S6-aptamer for specific delivery of miRNA-34a into NSCLC cells. S6 is a DNA aptamer, which was selected by an in vivo cell-SELEX method for lung adenocarcinoma cell appreciation [49]. The PAM-Ap/pMiR-34a nanocomplex selectively inhibited cell migration and proliferation, as well as induced apoptosis in an NSCLC in vitro model, compared with non-aptamer coated nanocarriers. MiR-34a indirectly activated *p57* expression and downregulated *Bcl-2* expression [50].

Discovered by Wang et al., the trans-RA16 (in vitro transcribed) aptamer specifically binds to lung cancer cells, which was discovered by in vivo selection. Its antiproliferative activity was significant in both in vitro and in vivo studies on NSCLC models. Aptamer binding specificity analysis on several cancer cell lines and a normal lung cell line showed the cancer type specificity of the RA16 aptamer for the NSCLC cell line. In the next stage, Wang et al. indicated that biotynylation of the 5′-end had no significant influence on the aptamer’s binding activity. They also compared the trans-RA16 aptamer with its chemically synthesized form (syn-RA16) and three truncated forms of syn-RA16. The binding affinity level was comparable for trans-RA16 and syn-RA16. Syn-RA16 internalized into tumor cells by receptor-mediated endocytosis. Only one truncated form, with an active 5′-end and 40 random nucleotides, had similar binding affinity as RA16 [51].

NAS-24 is an 80-nucleotide DNA aptamer selective against vimentin. Vimentin belongs to the type III intermediate filaments and forms microtubules, but its cellular functions depend on its post-translational modification and cellular location [52,53]. Vimentin is involved in intercellular contact and in the EMT process, thus affecting the ability of cancer cells to metastasize. High vimentin expression has been demonstrated in lung adenocarcinoma and large cell lung cancer [52,54]. In vivo studies on mouse lung adenocarcinoma models showed that NAS-24 stimulates the apoptosis of tumor cells and reduces tumor size [24]. Jalalian et al. created an ENPPASe complex consisting of selenium nanoparticles loaded with epirubicin and coated by two aptamers: NAS-24 and 5TR1 aptamers conjugated with PEI-PEG. An in vitro test was performed for human breast cancer model and murin colon carcinoma, where the ENPPASe complex showed significant tumor regression in mice compared to the control [55].

The MUC1-aptamer hybrid nanoparticle for specific delivery of regulatory miRNA-29b to NSCLC cells was discovered by Perepelyuk et al. miRNA-29B is the most profusely expressed miRNA in the miRNA-29 family and is downregulated by c-Myc. Low expression levels of miRNA-29b in cancer cells are associated with higher aggressiveness of the tumor. miRNA-29b is responsible for downregulation of DNA methyltransferase 3B (DNMT3B) in lung cancer cells [56,57,58]. The MUC1-aptamer hybrid nanoparticle consists of a poloxamer-188–polyoxyethylene–poluoxypropylene anionic triblock copolymer conjugated with human immunoglobulin G for “protection” from the host’s immune system. After combining the hybrid nanoparticles with a MUC1-specific aptamer, the size of this nanocomplex was approximately 595 nm and its zeta potential was +4.1 mV (without MUC1, it was −2.1 mV). The MUC1-aptamer hybrid nanoparticle activates cancer cell apoptosis and inhibits cell proliferation by downregulation of both *DNMT3B* and *MCL1* genes. The nanocomplex accumulates on the surface of MUC1-positive cancer cells and is distributed by endolysosomal lysis inside the cell. Complete release of transported miRNA-29b takes place at a pH value of 5.0. Cell specificity was detected for NSCLC lines but not for the normal lung fibroblast cell line. Apoptotic level was higher in MUC1-aptamer hybrid nanoparticle than in a lipofectamine-transfected miRNA-29b probe [58]. The inhibition of tumor growth was confirmed in lung tumor-bearing SCID mice [59].

Another example of an anti-MUC1-aptamer nanocomplex is genistein-miRNA-29b-loaded hybrid (GMLNH). The total release of genistein and miR-29b was observed before 80 h in pH value of 5 and was reduced in pH values of 6.5/7. The particle size of the MUC-1 labeled nanocomplex was about 598 nm and its zeta potential was about 4.2 mV. Intracellular uptake of aptamer nanocomplex was tested on A549 and MRC-5 (human fibroblast) cell lines. The intracellular level of GMLNH was higher for A549 cells, and the complex accumulated inside the endosomes and nuclei. Decreased levels of MCL-1 and DNMT3B, but not for PI3K and AKT proteins, were detected in the A549 cell line. GMLNH showed higher proapoptotic properties on the A549 cell line than miRNA-29b and genistein treatment [60].

Liu et al. created a programmable DNA nanoparticle for selective delivery of Rab26 siRNA to MUC1-positive NSCLC. The nanocomplex showed aptamer-dependent intracellular uptake and essential anticancer properties [61].

MA3 is an DNA aptamer for selectively binding MUC-1 with a K_d_ value of 38.3 nM. Hu et al. used MA3 for targeted delivery of intercalated doxorubicin to lung adenocarcinoma cell line A549 and MCF-7 breast cancer cells. Control tests using an MUC1- HepG2 cell line showed trace fluorescence signal after Apt-DOX treatment and intensive lightening for DOX-treated cells. Apt-DOX accumulated in the cytoplasm, while free DOX accumulated in cell’s nucleus [62].

Persistent luminescence nanoparticle (PLN) with mesoporous structure loaded with afatinib and coated with Map aptamer (anti-MAGE A3 SH-modified DNA aptamer) was developed for selective drug delivery into the MAGE-A3 positive tumors and optical diagnostics. The AFT-PLN@Map nanocomplex was tested on in situ and metastatic lung adenocarcinoma models. In vitro analysis showed an 0.3 μg/mL IC_50_ value for AFT-PLN@Map, where the loaded dose of afatinib was 45 ng/mL. An in vitro study also confirmed cellular phagocytosis of the nanocomplex and intracellular accumulation. In vivo tests on in situ carcinoma showed a significant decrease in tumor size after five weeks treatment (80 mm^3^ compared to 590 mm^3^ for untreated control). The aptamer-coated complex had a longer drug-release period, compared to the AFT-PLN nanoparticle. Intravenous injection was related to several side effects. AFT-PLN@Map was accumulated mainly in the tumor, but was also detected in the liver and renal tissue. A metastatic in vivo cancer model showed inhibition of metastatic process by AFT-PLN@Map, but no tumor size reduction [63].

AP-74 M-545 is a galectin-specific DNA aptamer. The galectin protein is involved in escape from immune surveillance and the T lymphocyte apoptotic process. This protein is a ligand for the CD45 receptor. Inhibition of their interaction restores T-cell immunity. Bioinformatic analysis of the aptamers 2D structure showed stem-loop structures on both 3′ and 5′ ends. An in vivo study on LL/2-bearing mouse lung cancer model depicted the highest accumulation of AP-74 M-545 in the tumor, but it was also present in the liver. The antitumor effect of AP-74 M-545 was tested on LL/2-bearing mice and NOD/SCID mice. The highest IL-2 blood level and highest tumor infiltration by CD4+ and CD8+ cells were detected only in syngeneic mice. It was also noted that AP-74 M-545 should be modified for longer retention time [64].

ApMAFG3F, apMAFG6F, and apMAFG11F are DNA-aptamers which specifically bind to musculoaponeurotic fibrosarcoma oncogene family protein (*MAFG*), which is engaged in chemoresistance in NSCLC and breast tumors. The role of MSAFG is downregulation of ROS (reactive oxygen species) concentration in the cell. Vera-Puente et al. showed that the high expression level of MAFG decreased overall survival time in patients with NSCLC with P value of 0.011. Statistical analysis was prepared for patients with NSCLC: 984 from TCGA database and 1035 patients from the Total Cancer Care Biorespiratory at the Moffit Cancer Center. Three aptamers selected by the SELEX method were tested on two NSCLC’ cell lines, one resistant to cisplatin (H23R) and one sensitive (H23S). The highest specificity was confirmed for apMAFG6F. In 25 nM dose, apMAFG6F decreased cell resistance with resistance index 2.63 vs. 2.0. Vera-Puente et al. observed a linear dose response relationship between cisplatin sensitivity, according to the production value of ROS in the resistant cell line treated with apMAFG6F. The same correlation was observed in decreasing cell viability. ApMAFG6F was generally accumulated in the cell nucleus [65].

The OPN-R3 aptamer is directed against osteopontin. Osteopontin belongs to the group of cytokines responsible for the migration, adhesion, and proliferation of fibroblasts [66]. Jin et al. revealed the relationships between the simultaneous expression of ostepontin, αvβ3 integrin, PIM-3 integrin, and the stage of lung cancer and the presence of metastasis in lymph nodes [67].

A-P50 is a 31-nucleotide RNA aptamer with high affinity to the NF-κB p50 sub-unit. The p50 sub-unit is an active transcription factor. Its blocking results in inhibition of NF-κB-mediated activation of DNA replication [25]. A-P50 has shown activity in restoring the sensitivity of cancer cells to doxorubicin in in vitro experiments on lung adenocarcinoma cell lines and in in vivo studies using a mouse model. Mi et al. demonstrated that the resistance mechanism to doxorubicin is involved in angiogenesis through the HIF-1alpha/VEGF pathway in NSCLC [68].

EpCAM is a transmembrane protein which is highly expressed on solid tumor cells [69,70,71]. Its level of expression on healthy epithelial cell surfaces is significantly low; hence, EpCAM is considered to be a cancer antigen. As the EpCAM level is extremely high on breast cancer cells, most published papers have focused on that type of cancer. Nevertheless, several research papers have underlined that the expression of EpCAM on circulating tumor cells may play an important role in the diagnosis and treatment of metastatic lung cancer. The FDA-approved CellSearch system is used for EpCAM-positive CTCs isolation. The EpCAM protein is involved in the regulation of the cell cycle by the c-myc kinases, cyclin D, and survivin protein pathways. Furthermore, EpCAM also plays an important role in intracellular interactions and cell motility [71]. At present, there exist three chimeric molecules of EpCAM conjugated with siRNAs: the EpCAM-AsiC, EpCAMiC, and EpCAM-survivin chimeras [70]. Pak et al. analysed the significance of EpCam expression on cancer cells in a group of 164 patients with NSCLC. In a group of adenocarcinoma patients, the overexpression of EpCAM was highly correlated with several clinical dates, but was lower than in squamous cell carcinoma (SCC) patients. The expression level of EpCAM did not have a significance influence on OC and DSF (disease-free survival) for both adenocarcinoma and SCC group. Liao et al. showed the higher efficacy of gencitinibe in inhibiting EMT through the HGF/cMET pathway. Analyses of apoptosis in vitro and blood levels of EpCAM-positive CTCs before and after chemotherapy treatment in two groups of patients (one treated with common chemotherapy and another treated with common chemotherapy as well as gemcitabine) showed that gemcitanibe specifically targeted EpCAM+ cells [72].

EpCAM-specific aptamer modification directions include aptamer conjugation with siRNA, bispecific aptamers, or aptamer–monoclonal antibody conjugation for selective drug delivery, overcoming the blood brain barrier (BBB), or labelling of nanocarriers.

The BBB can be overcome by utilizing active transport in epithelial cells. One of the key molecules is the transferrin receptor (TfR) [7]. MacDonald et al. developed a bifunctional aptamer consisting of an anti-EpCAM aptamer and a second anti-TfR aptamer. This bifunctional aptamer was then conjugated to DOX. Analysis of its BBB-overcoming ability was performed in a mouse model transfected with triple negative breast cancer cells metastasizing into CNS, MA-MB-231Br. Four molecule complexes were studied: TEPP (EpCAm–TfR bispecific aptamer), TEPP-DOX (EpCAm–TfR bispecific aptamer conjugated with doxorubicin), TENN (EpCAM–TEFF non-specific aptamer), and TENN-DOX (EpCAM–TEFF non-specific aptamer conjugated with doxorubicin). Brain penetration was observed with each complex. Accordingly, with published data, the poor lighting of the TENN and TENN-DOX complexes indicated non-specific transport of the complex through the BBB. The penetration of TEPP and TEPP-DOX into the CNS was significantly higher (TEPP-DOX > TEPP). The dose of doxorubicin in the aptamer complex was 2 mg/kg. Brain sections were prepared 60 min and 75 min (for complexes containing DOX) after intravenous administration. Attachment of doxorubicin with the aptamer significantly reduced the affinity of the anti-EpCAM aptamer, simultaneously increasing the affinity of TfR [73]. Alibolandi et al. formed the aptamer EpCAM (fluoropyrimidine RNA aptamer) in complex with PLGA-b-PEG nanopolymersomes loaded with doxorubicin. In vitro tests were prepared on NSCLC cell lines SK-MES-1 and A549 and in vivo studies were carried out in mouse xenograft models transfected with SK-MES-1 cells. The effectiveness of cell poriferans inhibition was twice higher in the aptamer nanocomplex with DOX than with doxorubicin monotreatment [74].

Engelberg et al. developed S15-APT-PEG-PCL nanocarriers loaded with small doses of paclitaxel (PTX) targeted at the human adenocarcinoma A549 cell line. The obtained nanocarriers revealed a selective cytotoxicity for the A549 cell line. The binding selectivity of aptamer-labelled nanocarriers was tested against two other solid tumor cell lines and four normal cell lines, including human bronchial epithelial BEAS2B cells. The half-maximal inhibitory concentration value was 0.03 μM. The size of the S15-APT-PEG-PCL nanoparticle was about 15 nm, in the ideal size range for therapeutic nanoparticles. The research group also revealed that the selective enhancing of tumor cells depends on S15 aptamers. The cell internalization mechanism of S15-APT-PEG-PCL nanoparticles loaded with PTX operated by clathrin-mediated endocytosis. The chemical structure of this nanocarrier allows easy and long-term release of the chemotherapeutic agent inside the target cell. The core of the NPs is polycaprolactone (PCL) conjugated with hydrophilic polyethylene-glycol (PEG) and coated with 30 nM of S15-aptamers. After endocytosis, PTX loaded S15-APT-PEG-PCL NPs undergo enzymatic lysis by nucleases and lipases, and the hydrophobic drug paclitaxel is secreted inside the NSCLC cell. The S15 aptamer is a Cy5-labeled 85-nucleotide DNA aptamer, which was commercially synthesized [75].

NOX-A12 is an L-RNA aptamer which inhibits stromal cell-derived factor-1 (SDF-1) function. SDF-1 is also called CXCL12 (C-X-C motif chemokine ligand 12), and is produced by tumor stromal fibroblasts (among others) [76]. It may indirectly participate in the modification of intercellular connections conducive to the EMT process [77]. In cancer, it is involved in creating an environment for metastasis [78]. In healthy people, CXCL12 is responsible for hematopoiesis and the recruitment of T lymphocytes to lymph nodes and the spleen [9]. In in vivo studies in an animal model, CXCL12 blockade via NOX-A12 resulted in increased macrophage recruitment to the tumor microenvironment and increased sensitivity to anti-VEGF antibody treatment [44]. NOX-A12 has a synergistic effect with PD-1 inhibitors. In animal models, the use of this aptamer together with immune checkpoint inhibitors revealed its effectiveness in cancer treatment without significant toxicity [76].

NOX-D12 is an L-RNA aptamer which inhibits the interaction of C5a with its receptor C5aR. The 5′-end of NOX-D12 is covalently linked, by an aminohexyl linker, with a 40 kDa polyethylene glycol moiety. The K_d_ of NOX-D12, as detected by plasmon resonance, for human C5a was 776 ± 9.5 pM. Twenty-eight days after intraperitoneal injection of NOX-D12 in NMRI mouse, plasma levels of the aptamer were at 21 ± 6.1 nM. The injection dose of NOX-D12 was 10 mg/kg. In vivo tests revealed a significant decrease of MDSC population without sensitive changes in lymphocyte T population after NOX-D12 treatment. Ajona et al. used combinational therapy of NOX-D12 and anti-PD-1 monoclonal antibody in a 393P mouse model. Combined therapy showed an essential decrease in tumor size, compared to two (untreated and treated by monotherapy) mice. After 42 days, the rejection of tumor in the group treated with the aptamer and mAb was confirmed. The tumor growth inhibition effect of combinational therapy was mediated by the activation of CD8 lymphocytes [79]. Ajona et al. also confirmed the important role of high expression levels of CXCL16 mediated by C5a/C5aR1 in bone-metastatic NSCLC mediated by C5a/C5aR1. NOX-D12 inhibited the metastatic process in a mouse model by binding to C5a [80].

Apt–PD-L1 is a 45-nucleotide DNA aptamer specific to PD-L1 (programmed death 1 ligand). The PD-L1 molecule is present on the surface of human cells, where its expression on cancer cells and immune cells which infiltrate the tumor microenvironment is particularly high. The action of the PD-L1 molecule on cancer cells with the PD-1 receptor on cytotoxic and helper T cells results in their anergy [7,81]. Blockade of the PD-L1/PD-1 interaction is one of the mechanisms of immunotherapy action in cancer patients, which consists of restoring the activity of cytotoxic T lymphocytes for the development of an anti-tumor response. In in vivo studies on mouse models with lung cancer, a significant decrease in tumor size was observed during apt–PD-L1 treatment. Increased recruitment of cytotoxic and helper T lymphocytes into the tumor microenvironment was also confirmed. In addition, significantly higher levels of CXC9 and CXC10 chemokines were detected in the serum of mice receiving this aptamer, which are involved in the inhibition of tumor neovascularization [82].

The anti-CTLA4 DNA aptamer significantly stimulated the activation of T lymphocytes for antitumor defense in in vitro and in in vivo studies [83]. CTLA4 (cytotoxic T lymphocyte-associated protein 4) belongs to immune checkpoints and is responsible for the inhibition of excessive activation of T lymphocytes recruited in lymph nodes [84]. In cancer, it acts as an inhibitor of the immune response to tumor cells. CTLA4 belongs to the family of receptors for CD80 and CD86 molecules. It displaces the CD28 molecule on lymphocytes from binding to the CD80 and CD86 molecules present on antigen-presenting cells [85,86]. Soldevilla et al. created a bispecific aptamer which binds simultaneously to CTLA4 and to ICOS (inducible T cell costimulator). ICOS belongs to immune checkpoints and transmits another signal for lymphocyte inactivation. In vitro and in vivo studies in a mouse model showed T cell activation and their anti-tumor response to be induced by the anti-CTLA4 aptamer [86].

Apt8a is an agonist aptamer specific to ICOS. In vivo tests showed no significant changes in the melanoma tumor after intravenous injection. Nevertheless, intratumor injection showed a significant decrease in tumor size. The Apt8a-antiMRP1-bispecific aptamer delivered into the tumor and the intravenous injection of anti-CTLA4 monoclonal antibodies involved the highest tumor infiltration by lymphocyte T [86]. MRP1 is expressed in cancer stem cells and may play an important role in drug resistance mechanisms.

## 4. Conclusions

Aptamers have recently become useful tools for developing novel therapeutics. In technical terms, we can distinguish two strategies for developing new anti-NSCLC aptamers: first, the selection process takes place in vivo and new aptamers specific to cancer cell types are developed. Second, the selection is prepared based on known molecules. This second strategy gives us the possibility not to discover potential anticancer agents, but also provides us with new information about biological processes. At present, the dominant direction in the development of aptamer technologies is the creation of bispecific aptamers or their use in combined therapy with other drugs; that is, targeted molecular therapies or immunotherapy. A particularly important trend in scientific research in recent years is nanotechnological innovation. According to the presented data, aptamers help to make nanoparticles less immunogenic and more specific, whereas nanoparticles prolong the half-life of aptamers in plasma and moderate their clearance. Aptamers in complex with chemotherapeutic agents can serve to eliminate the severity of their side-effects by lowering (or eliminating) their toxicity in normal tissue. Promising treatment strategies might be provided by the combination of nanocarrier and chemotherapeutic drugs labeled by aptamers. These kind of particles can provide a reduced dose of drug, allowing for its release inside the tumor to be more prolonged and more specific. In effect, aptamer-labeled nanocarriers loaded with drugs may be less toxic and more efficient than traditional chemotherapy agents. Another interesting strategy is chimeric particle synthesis, which mainly consists of gene therapy linked with targeted aptamer therapy. Both nanoparticles as well as chimera molecules require more research on their safety. However, the production cost of these kinds of therapies may be high, hence limiting access to them. The most plausible and fast way for their introduction into clinical practice seems to be conjugation of aptamers with chemotherapeutic agents and already-used monoclonal antibodies. In this way, we can obtain a better version of a well-known molecule and the extent of clinical trials may be shortened. Conjugation of aptamers with chemotherapeutics or already-used TKI inhibitors can restore tumor drug sensitivity. Bifunctional molecules, consisting of immunotherapy drugs, aptamers, or the simultaneous use of both, may speed up a positive response to treatment. In technical aspects, these kinds of bifunctional molecule can address the main disadvantages of aptamers as therapeutic agents. The state of knowledge necessary to develop an effective aptamer-based drug is enormous. The key point to rapid introduction into therapeutic strategies is the appropriate application of aptamers. Based on collected knowledge, aptamers might be used as an “intelligent application” for already-used therapeutics.

## Supplementary Materials

The following are available online, Table S1: Clinical trials with selected aptamers mentioned in this article (I—first phase clinical trial, II—second phase clinical trial, III—third phase clinical trial, (-)—no data) [87].
