# Supplementary material for: Aptamers in Non-Small Cell Lung Cancer Treatment
Source: Molecules. 2020 Jul 9;25(14):3138. doi: 10.3390/molecules25143138 (PMC7396979; doi:10.3390/molecules25143138)
Supplement: Supplementary file 1 [file molecules-25-03138-s001.pdf]

Review

Aptamers in Non-Small Cell Lung Cancer Treatment

Irena Wieleba *, Kamila Wojas-Krawczyk and Paweł Krawczyk

Department of Pneumonology, Oncology and Allergology, Medical University of Lublin, 20-090 Lublin, Poland; kamilawojas@wp.pl (K.W.-K.); krapa@poczta.onet.pl (P.K.)

* Correspondence: i.m.wieleba@gmail.com

Received: 5 June 2020; Accepted: 8 July 2020; Published: date

Abstract: Aptamers are short, single-stranded oligonucleotides which are capable of specifically binding to single molecules and cellular structures. Aptamers are also known as "chemical antibodies". Compared to monoclonal antibodies, they are characterized by higher reaction specificity, lower molecular weight, lower production costs, and lower variability in the production stage. Aptamer research has been extended during the past twenty years, but only Macugen® has been accepted by the Food and Drug Administration (FDA) to date, and few aptamers have been examined in clinical trials. In vitro studies with aptamers have shown that they may take part in the regulation of cancer progression, angiogenesis, and metastasis processes. In this article, we focus on the potential use of aptamers in non-small cell lung cancer treatment.

Keywords: aptamers; NSCLC; cancer

Table 1. Clinical trials with selected aptamers mentioned in this article (I – first phase clinical trial, II – second phase clinical trial, III – third phase clinical trial, (-) – no data).

Aptamer's name	Molecular targets	Diseases	Clinical trials
Pegaptanib	VEGF	AMD<br>Diabetic Macular Oedema<br>PDR	NCT00321997 (II, III)<br>NCT00312351 (IV)<br>NCT00040313 (II)<br>NCT01487044(-)<br>NCT01487070 (I)
E10030<br><br><br><br><br><br>ARC 126<br>AX 102	PDGF	AMD<br><br><br><br><br><br>AMD<br>AMD	NCT01089517 (II)<br>NCT00569140 (I)<br>NCT01944839 (III)<br>NCT01940900 (III)<br>NCT01940887 (III)<br><br>pre-clinical phase<br>pre-clinical phase

NOX-A12	SDF-1	Autologous Stem Cell Transplantation Hematopoietic Stem Cell Transplantation	NCT00976378 (I) NCT01194934 (I)
AS1411	nucleolin	Acute Myeloid Leukemia	NCT01034410 (II)
E0727/CL428/KD1130/TuTu223 A1532/4233/CL434/CL4	EGFR	Solid tumors	pre-clinical phase
NAS-24	Vimentin	Solid tumors	pre-clinical phase
OPN-R3	Osteopontin	Bone metastases	pre-clinical phase
A-P50	NF-κB	Solid tumors	pre-clinical phase
Apt-PD-L1	PD-L1	Solid tumors	pre-clinical phase
Anti-CTLA4	CTLA4	Solid tumors	pre-clinical phase
